# Identifying Human Genome-Wide CNV, LOH and UPD by Targeted Sequencing of Selected Regions

**DOI:** 10.1371/journal.pone.0123081

**Published:** 2015-04-28

**Authors:** Yu Wang, Wei Li, Yingying Xia, Chongzhi Wang, Y. Tom Tang, Wenying Guo, Jinliang Li, Xia Zhao, Yepeng Sun, Juan Hu, Hefu Zhen, Xiandong Zhang, Chao Chen, Yujian Shi, Lin Li, Hongzhi Cao, Hongli Du, Jian Li

**Affiliations:** 1 School of Bioscience and Bioengineering, South China University of Technology, Guangzhou, China; 2 BGI-Shenzhen, Shenzhen, China; 3 Weifang Hospital of Traditional Chinese Medicine, Weifang, Shandong, China; 4 Complete Genomics, Inc., 2071 Stierlin Court, Mountain View, California, 94043, United States of America; 5 School of Life Sciences, Sun Yat-sen University, Guangzhou, China; 6 University of Copenhagen, Department of Biology, Copenhagen, Denmark; Hospital Authority, CHINA

## Abstract

Copy-number variations (CNV), loss of heterozygosity (LOH), and uniparental disomy (UPD) are large genomic aberrations leading to many common inherited diseases, cancers, and other complex diseases. An integrated tool to identify these aberrations is essential in understanding diseases and in designing clinical interventions. Previous discovery methods based on whole-genome sequencing (WGS) require very high depth of coverage on the whole genome scale, and are cost-wise inefficient. Another approach, whole exome genome sequencing (WEGS), is limited to discovering variations within exons. Thus, we are lacking efficient methods to detect genomic aberrations on the whole genome scale using next-generation sequencing technology. Here we present a method to identify genome-wide CNV, LOH and UPD for the human genome via selectively sequencing a small portion of genome termed Selected Target Regions (SeTRs). In our experiments, the SeTRs are covered by 99.73%~99.95% with sufficient depth. Our developed bioinformatics pipeline calls genome-wide CNVs with high confidence, revealing 8 credible events of LOH and 3 UPD events larger than 5M from 15 individual samples. We demonstrate that genome-wide CNV, LOH and UPD can be detected using a cost-effective SeTRs sequencing approach, and that LOH and UPD can be identified using just a sample grouping technique, without using a matched sample or familial information.

## Introduction

Copy-number variations (CNV)[1]and loss of heterozygosity (LOH)[2] are different types of genomics aberrations. CNV is defined as a variation from the reference genome by a more than 1Kbp DNA segment, either via duplication or deletion[3]. LOH is manifested by unusual long stretches of homozygous SNPs. When a LOH occurs without a change in copy number(CN), i.e. that both copies are inherited from only one parent, it is called copy-neutral LOH, or uniparental disomy (UPD)[4,5]. CNV, LOH, and UPD are important factors leading to many common inherited diseases, cancers, and other complex diseases[6–10]. Thus, accurately identifying genome-wide CNV, LOH and UPD is essential in understanding diseases and in designing correct clinical interventions.

For a long time, SNP genotyping arrays[11]and array Comparative Genomic Hybridization (aCGH)[12] have been deemed as standard means to detect CNV or LOH. Those DNA microarrays, however, suffer some common limitations—most notably that the measured CN ratio from fluorescence intensities is noisy[13–16] and the experimental results require further examination from an experienced person.

With the rapid decrease in price and increase in accuracy with next-generation sequencing (NGS), more and more CNV and LOH studies are turning to NGS. Four methods for genome-wide CNV detection have been established recently based on whole-genome sequencing (WGS) using NGS. There are: paired-end mapping, read-depth analysis, split-read strategies, and sequence assembly comparisons[17–20]. These methods require high depth of coverage on whole genome scale. Other approaches with low coverage depth on WGS cannot detect heterozygous positions for LOH and UPD[21,22]. Another parallel method is to sequence only the exome. The exome-only method detects CNVs associated with exons and typically small in size (~100—200bp). Their distribution in the genome is uneven. Thus, exome-only sequencing fails to capture a global picture of genome-scale aberrations.

A limited number of approaches have been developed for small CNV or LOH analysis using target region(TR) sequencing[23,24]. The current TR-approaches in practice are also limited to detect variations involving one or a few of exons. Most methods, based on TR sequencing, eliminate bias (GC bias etc.) by using some correction methods; but it is known that some local variations in depth-of-coverage cannot be removed by the GC-based correction[25] and the non-contiguous nature of target regions poses a different challenge to computational methods. For example, longer genes are on average better covered compared to shorter ones; and low-complexity target regions usually have poor coverage. Further, most of them do not discriminate between two- and single-copy deletion and between three- and multiple-copy amplifications. They cannot predict exact copy number of a genomic segment and fail to identify large LOH and UPD without a matched control or family members.

**Fig 1:**
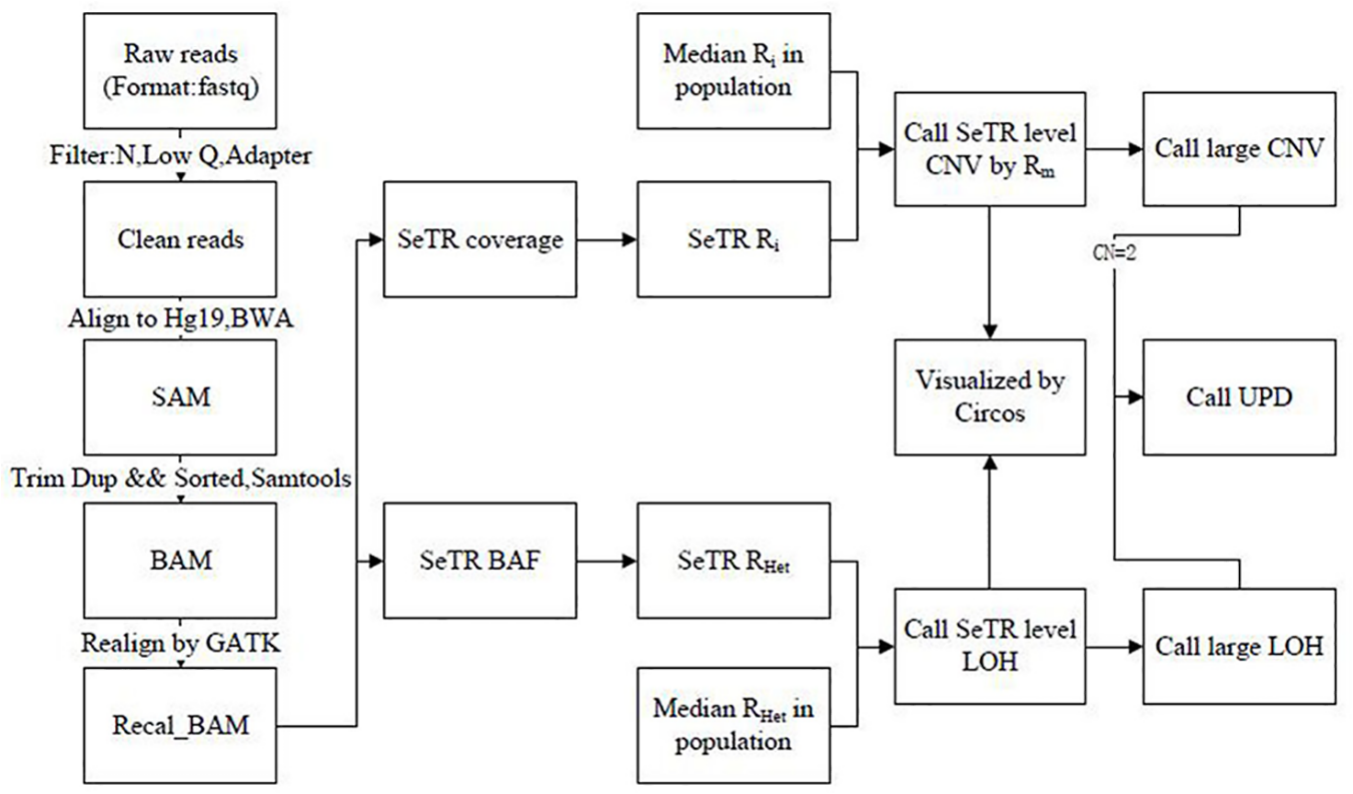
Overview of the ICLU pipeline. The pipeline takes the raw FASTQ files or the aligned BAM files as input, and outputs the genome-wide CNV, LOH and UPD results with visualization.

In summary, so far no method has been proposed to avoid the defects of WGS and TR sequencing in identifying all genome-wide CNV,LOH and UPD without a matched sample. To address this issue, we elaborately designed a special genome-wide segmental partition termed Selected Target Regions (SeTRs). SeTR is composed of evenly distributed small SNPs and short random repeat markers and it collectively covers 1.46% of the whole genome (2.86G bp, hg19). The average length of SeTRs’ probes is ~150bp and the median physical distance between two adjacent probes is about 10.6kb. We also established a bioinformatics pipeline named ICLU (Identifying genome-wide CNV, LOH and UPD). ICLU employs T-test to detect CNV using the depth-of-coverage of targeted regions and employs F-test to call LOH using heterozygous coefficient of polymorphic position. It combines CNV and LOH to infer UPD, and visualizes genome wide alterations via Circos[26] (Fig 1). We used simulation data as well as real samples with known variations to validate our method. We applied our method to detect genomic-wide aberrations in 15 real samples. By grouping samples together, we are able to achieve variation detection without using a matched sample or familial information. One shortcoming is that TR sequencing cannot resolve novel, small variants (SNPs and indels) located within the designed target regions. Aside from this minor problem, we believe TR sequencing technology has great potential for studying genome-wide CNV, LOH, and UPD.

## Results

### Evaluation of SeTR Sequencing

In this study, we have designed 278,800 probes that are small DNA segments selected from the published human reference genome, Build 37.1, hg19. The total size of the probes is 41,795,106bp(~42Mb). Our probes cover 1.46% of the whole effective genome (2.86G bp, hg19). The average length of probe is about 150bp and the median physical distance between two adjacent probes is about 10.6kb genome wide (S1 Table and S1 Fig). We also vindicated the distribution of SeTR probes on three real samples before the downstream analysis.

Three sequence libraries were generated from genomic DNA (gDNA) of three samples, including two normal samples (YH and HG00537) and a Coriell Institute sample, GM50275, known to contain a positive CNV. The three libraries were then sequenced via the Illumina high throughput sequencing platform. After filtering out reads with low sequencing quality scores (Q<20)[27] or with adapters’ sequence, the clean data was mapped to the human genome reference assembly (Build 37.1, hg19). 66.93%-67.87% of clean reads were aligned to target regions, representing 95.16%-97.09% of the uniquely mapped. Under the condition that the mean target region coverage was 70 reads or above, the alignment results showed that 99.73%-99.95% of the target regions were covered by at least one reads and over 99% by at least ten reads (Table 1). This aligned coverage of target regions was better in evenness than the coverage from other capturing methods, such as exome capturing, with similar mean coverage[28].

**Table 1:** Data production and mapping results for the three samples used.

Sample	YH	HG00537	GM50275
**Target region(bp)**	41,795,106	41,795,106	41,795,106
**Raw reads**	78,544,670	77,826,604	80,181,866
**Raw data(Mb)**	7,067.69	7,003.09	7,211.26
**Clean reads**	66,288,119	63,136,026	62,010,469
**Clean data (Mb)**	5,965.93	5,682.24	5,580.94
**Clean reads mapped to genome (%)**	99.29	98.13	98.14
**Clean reads uniquely mapped to genome (%)**	97.09	95.96	95.16
**Clean reads mapped to target region (%)**	67.43	66.93	67.87
**Mean depth of target region (X)**	70.89	68.15	67.3
**Coverage of target region (%)**	99.94	99.73	99.95
**Fraction of target covered > = 4X (%)**	99.9	99.52	99.89
**Fraction of target covered > = 10X (%)**	99.48	99.19	99.44
**Fraction of target covered > = 20X (%)**	96.65	96.57	96.34
**Fraction of target covered > = 30X (%)**	90.02	89.79	88.99
**Fraction of target covered > = 40X (%)**	80.03	79.01	77.81

**Fig 2:**
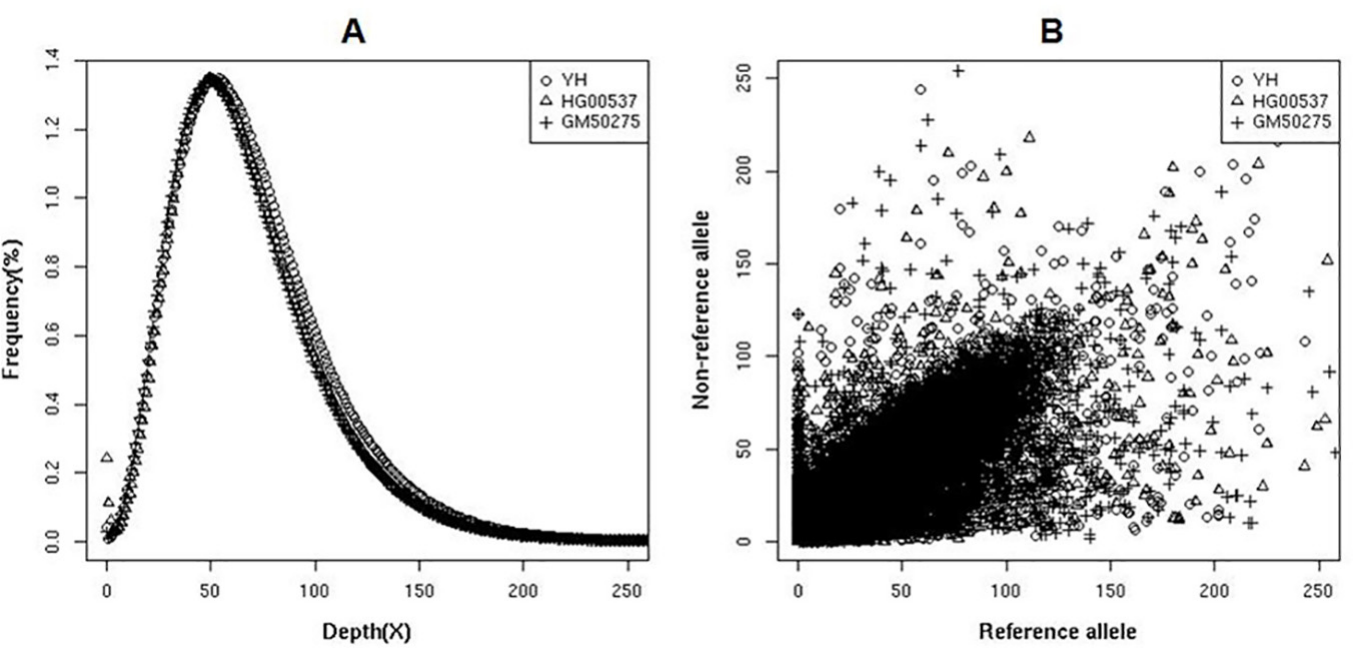
Characteristics of SeTRs in three real samples. (A) Distribution of coverage depth in SeTR; (B) The distribution of supported non-reference and reference allele reads at SNPs’ sites.

The coverage depth distribution of target regions showed a similar Poisson distribution for all three samples, indicating an even enrichment of the target regions (Fig 2A). Most SNPs’ sites called by GATK software have similar support reads for the non-reference allele and for the reference allele, inferring good enrichment balance for the two haplotypes (Fig 2B).

### Characteristics of depth-of-coverage and heterozygous coefficient in SeTRs

**Fig 3:**
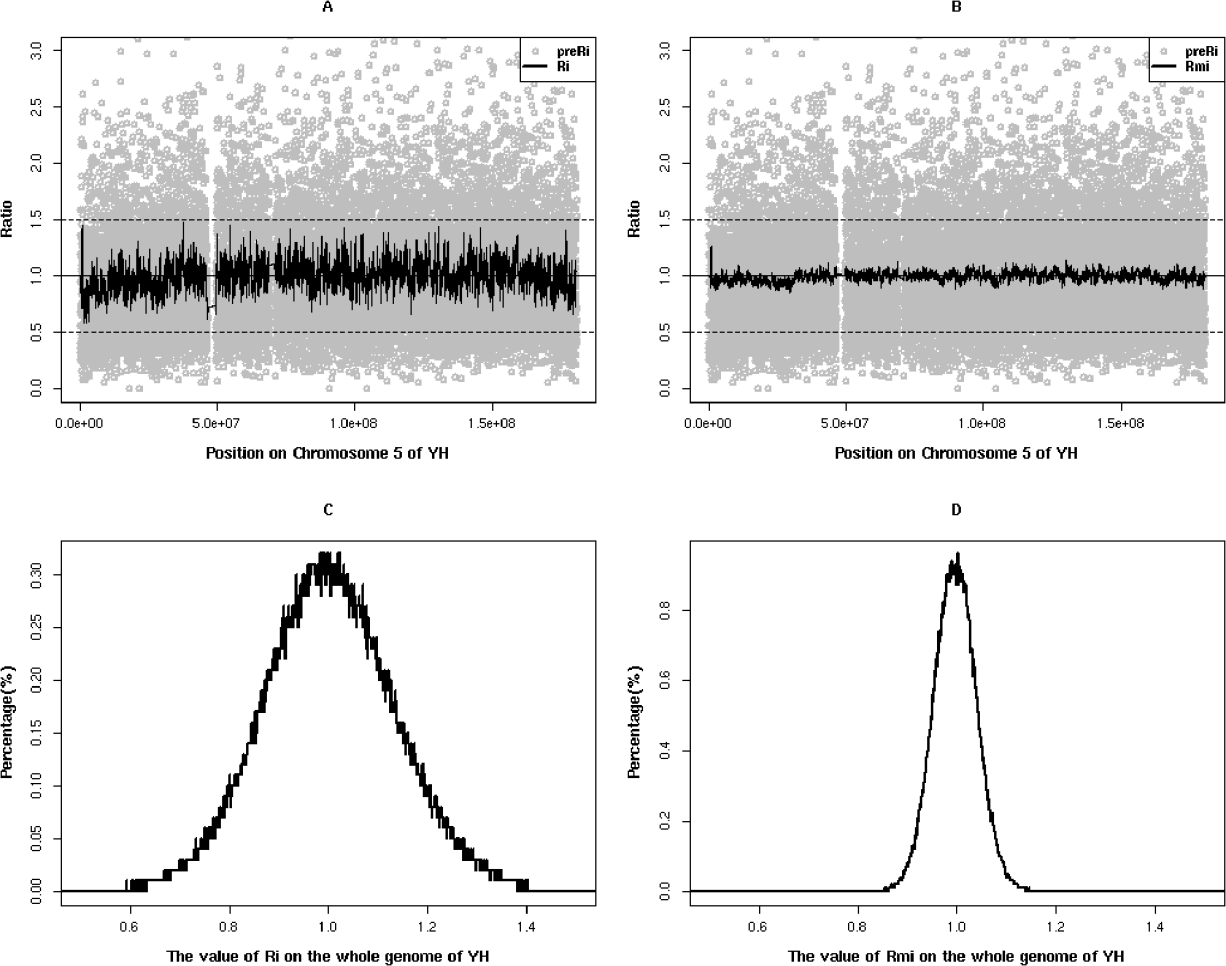
Characteristics of three ratios in YH sample. (AB)The distribution of three ratios across Chromosome 5.The imaginary line (Ratio = 0.5) means the CN equals to 1 and the imaginary line (Ratio = 1.5) CN equals to 3. After smoothing and renormalized steps, the fluctuation of ratios decreased gradually from preR_i_ (gray circle points) to R_i_, and then to R_mi_ (black line). (C)The distribution of R_i_ in the whole genome;(D)The distribution of R_mi_ in the whole genome.

**Fig 4:**
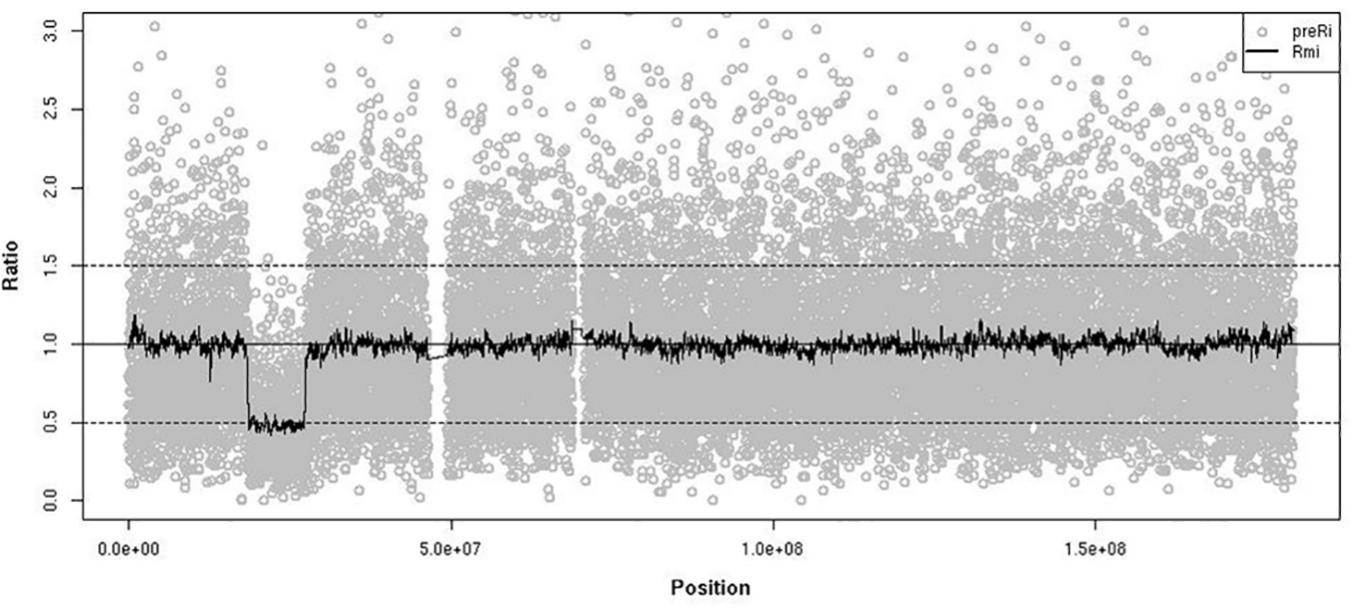
Characteristics of preR_i_ and R_mi_ on Chromosome 5 of GM50275 individual.

To detect CNV, the depth-of-coverage of SeTRs was calculated from the re-corrected alignment results and then was transformed to preR_i_ by dividing its coverage depth by the average depth of all target regions for the sample(see Methods). We found that this preR_i_ has large fluctuations on the whole genome scale, which is expected due to the characteristics of each target region and the different capture efficiency of the probes (Fig 3AB). In order to keep the relative stability of the fluctuations in contiguous target regions, two correction strategies were applied:1) We selected the mean value of ten downstream target regions’ depth (TD_mi_) of the target *i* region to replace TD_i_ to get depth coefficient (R_i_) using a smoothing fit. 2) We generate R_m_ by dividing R_i_ with the geometric median of all R_i_s of in the same target i region in multiple samples. The median of R_i_, regarded as a robust baseline to reduce the adverse effect of experimental conditions and capture efficiency, is essential to renormalize R_i._ A few of R_i_s alone in normal samples failed to be normalized to 1 by formulas (1,2,3,4,5) (see Methods)(Fig 3A). After those smoothing and renormalization steps, the final corrected ratio (R_mi_) showed much smaller variability across the whole genome. It is much closer to the normal distribution with a mean of 1(from 1.207 to 0.959) and a smaller standard deviation (from 0.54 to 0.29) than R_i_ (Fig 3) in YH. When using the above approach to analyze the depth-of-coverage of SeTRs on chromosome 5 of GM50275 individual, a copy number loss event (del(5)(p14)) gradually emerged (Fig 4), consistent with the known and confirmed result (Table 2).

**Table 2:** The detected results of genome-wide CNV of 15 confirmed samples.

Sample	Confirmed	ICLU(~42Mb SeTRs)		ICLU(~5Mb SeTRs)	
	CNV	CNV	CN	CNV	CN
**YH**	46,XY	46,XY	2	46,XY	2
**HG00537**	46,XX	46,XX	2	46,XX	2
**GM50178**	46,XX,del(5)(p15.3)	46,XX,del(5)(p15.3)	1	46,XX,del(5)(p15.3)	1
**GM50275**	46,XY,del(5)(p14)	46,XY,del(5)(p14)	1	46,XY,del(5)(p14)	1
**GM12959**	46,XY,del(1)(q43)	46,XY,del(1)(q43q44)	1	46,XY,del(1)(q43q44)	1
**GM11419**	49,XYYYY	49,XYYYY	4	49,XYYYY	4
**GM22364**	46,XY,dup(15)(q11q12)	46,XY,dup(15)(q11q12q13.1)	3	46,XY,dup(15)(q11q12q13.1)	3
**GM05047**	46,XY,dup(10)(q11.2q23.2)	46,XY,dup(10)(q11.2q23.2)	3	46,XY,dup(10)(q11.2q23.2)	3
**GM50142**	46,XY,dup(18)(q21.2q22)	46,XY,dup(18)(q21.2q22)	3	46,XY,dup(18)(q21.2q22)	3
**GM12074**	46,XY,del(16)(q22q23)	46,XY,del(16)(q22q23)	1	NA	NA
**GM10922**	46,XY,del(3)(p25)	46,XY,del(3)(p25p26)	1	NA	NA
**GM10932**	46,XY,del(8)(p23)	46,XY,del(8)(p23)	1	NA	NA
**GM03623**	48,XXX,+18	48,XXX,+18	3,3	48,XXX,+18	3,3
**GM05875**	46,XX,del(16)(p12p11.2)	46,XX,del(16)(p12p11.2)	1	46,XX,del(16)(p12p11.2)	1
**GM08696**	46,XY,dup(18)(q21.3q12.1)	46,XY,dup(18)(q21.3q12)	3	46,XY,dup(18)(q21.3q12)	3

Note: “NA” means there is no result due to failing to make a NGS library.

**Fig 5:**
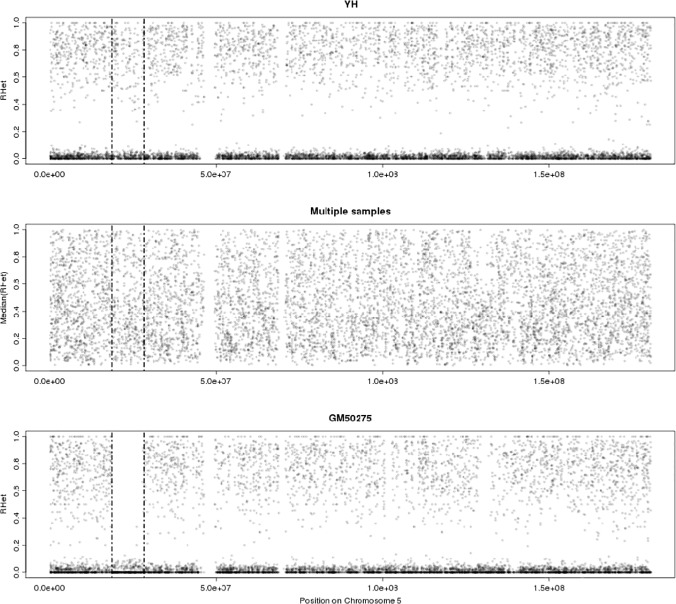
The distribution of R_Het_ across Chromosome 5 in YH, multiple samples and GM50275. (A) R_Het_s for the normal sample, YH; (B) Median R_Het_s for multiple samples; (C) R_Het_s for the positive sample,GM50275.

To estimate LOH, polymorphic positions with high allele frequency between 0.1 and 0.9 in the 1000 Genome SNPs Database (ftp://ftp.ncbi.nih.gov/1000genomes/ftp/release) in SeTRs of samples were retained and the non-reference-allele or “B-allele” frequency (BAF) of these positions was substituted by heterozygous coefficient (denoted as R_Het_, see Methods) to represent the heterozygous status of these local sites in SeTRs. In order to eliminate the individual background difference and give reasonable expression of R_Het_, median R_Het_ was introduced. It is the geometric median of all R_Het_s for every polymorphic position in the collection of multiple samples. By R_Het_’s definition, if a LOH occurs in a sequenced region, the expected sets of R_Het_s on the sequenced regionequal0 and otherwise they should equal1. In practice, most of R_Het_s or median R_Het_s were distributed between 0 and 1 across the whole chromosomes in one normal sample or in multiple samples (Fig 5). PCR amplification bias in NGS[29] may cause a haploid fragment pairs not equal in amounts. In our investigation, on chromosome 5p14 in GM50275 individual, a loss event happens, the sets of R_Het_s were close to 0 (Fig 5) and it reveals obviously that there was a LOH. Based on this reasoning, an F-test is applied in our method to detect significance increases in variance of R_Het_s of a genomic region in a test sample from that of median R_Het_s in the collection of multiple samples (see Methods).

### The performance of ICLU

We first used simulated data and then real samples’ data to assess the accuracy and power of our method for detecting genome-wide CNV. As our first step, we applied ICLU and CONTRA developed on WEGS[24], to detect small CNV with sizes ranging from 450Kb to 3Mb, and to identify the boundaries (break point detection) using the simulated whole genome sequencing data. With the same SeTRs, we simulated the Illumina paired-end (PE) reads with ~30X coverage of 8 individual samples using wgism (website:https://github.com/lh3/wgsim) but only performed the simulation on Chromosomes 19 and 20 of hg19 because of limited computing resource. The simulated sequence data has a median insert size of 200bp and a read length of 100bp. 3 of 8 individual samples are designed as true positive CNV samples. The other 5 samples are designed as normal, and are used as a control set so as to create a robust base line. All these simulated data received CNV analysis using ICLU pipeline described above (Fig 1) with parameters-M 10,-P 0.05 and CONTRA with default parameters. ICLU analysis results captured all 9 true positive events containing CN, and no false positive with 100% of sensitivity and 100% of specificity(S2 Table and S2 Fig). In comparison, CONTRA reports 11 CNV events, 8 are true positive and 3 false positive, thus behaving with 88.9% of sensitivity and 66.7% of specificity(S2 Table)

In the second step, we applied ICLU on 55X~90X of SeTRs sequencing data of 15 real human individuals, including 2 normal samples and 13 samples with true positive CNV events, all of which have been studied before. A robust base line of median R_i_ was constructed from 15 samples, and all samples were searched for CNVs over 1Mb at the p-value of 0.05 with the minimal number of probes setting at 45 or the minimal size of region at ~0.5Mb (45*~10kb = ~450kb). In total, 13 out of 15 test samples were identified with CNVs over 4Mb or with aneuploidies, including 11 events of CNVs from 11 samples and 3 aneuploidies from 2 samples. Among those, 7 events were single-copy deletions, 6 events three-copy amplifications, and 1 event a four-copy amplification on chromosome Y. In summary, the CNV results estimated by ICLU were highly consistent with confirmed CNV results (Table 2). The results demonstrated that ICLU in this case presented 100% sensitivity and 100% specificity(S3 Table).

We also studied the ability of our method at different coverage depth of SeTRs by gradually decreasing the depth of SeTRs from 55X~90X to 5X. The performance of ICLU did not degenerate significantly as coverage depth decreases. Almost all known CNV were discovered with no false positive predictions (S4 Table), even at its lower depth level of 8X.If coverage is below 8X,CNV calls by ICLU are no longer reliable (S3 Fig). There is one exception concerning an aneuploidy prediction on chromosome Y of sample GM11419 with 30X average coverage depth. Its computed mean CN is 3.497, giving a false predicted CN of 3 after round off (whereas the correct CN should be 4). In this case, the density of probes on chromosome Y is not high enough (S1 Table) to keep R_mi_ stable with lowered average coverage. This problem can be fixed by increasing the probe density at this region without raising coverage depth, or, of course, by increasing the average coverage depth as was shown before.

We also used ICLU to analyze LOH and UPD events within these 15 real samples on 55X~90X depth-of-coverage of SeTRs sequencing data. 8 events of LOH, whose sizes are larger than 5Mb, were observed under the p-value of 0.01; and all boundaries of LOH (CN = 1) were consistent with CNV results. Furthermore, combining with CNV (CN = 2) and LOH results, 3 isodisomy events of UPD were identified (Table 3). Without their familial information or matched samples, we cannot confirm the accuracy of these findings. But at least in theory, when CN was equal 1, LOH should happen, and that was captured in our results.

**Table 3:** The detected results of genome-wide LOH and UPD in15 test samples.

Sample	Chromosome	Start	End	Size(>5M)	LOH	CN
**YH**	-	-	-	-	-	-
**HG00537**	-	-	-	-	-	-
**GM50178**	chrX	103489643	108870605	5.38	UPD	2
	chr5	38139	5893356	5.86	LOH_nonUPD	1
**GM50275**	chr5	18601469	28281734	9.68	LOH_nonUPD	1
**GM12959**	chr1	242808483	248553940	5.75	LOH_nonUPD	1
	chr10	38160098	43475568	5.32	UPD	2
**GM11419**	chr3	46077525	51871405	5.79	UPD	2
**GM22364**	-	-	-	-	-	-
**GM05047**	-	-	-	-	-	-
**GM50142**	-	-	-	-	-	-
**GM12074**	chr16	67747306	75697469	7.95	LOH_nonUPD	1
**GM10922**	chr3	75084	11736290	11.66	LOH_nonUPD	1
**GM10932**	-	-	-	-	-	-
**GM03623**	-	-	-	-	-	-
**GM05875**	-	-	-	-	-	-
**GM08696**	-	-	-	-	-	-

Note: “LOH_nonUPD” means there is a LOH, but not UPD; “-” means there is no LOH events in this sample.

Moreover, we redesigned another smaller SeTRs set according to the same designing approach as described in Methods, the total size of which is 4,926,646bp(~5Mb).We used ICLU to analyze the above cell-line samples and it is demonstrated that the ICLU based on this SeTRs(~5Mb) has as good performance in detecting CNVs as that based on SeTRs(~42Mb)(Table 2 and S3 Table). This indicates that ICLU is flexible with its number of probes, and the results produced by ICLU are reproducible even though the SeTR probes are significantly reduced. Of course, the resolution power on CNV boundaries will drop as number of probes are decreased systematically. We also tested ICLU algorithm on 5 samples from aborted fetuses with unknown result and then validated these predictions by WGS method[30]. The data showed that ICLU, just as the WGS approach, can produced highly reliable results (S5 Table).

### Visualization

**Fig 6:**
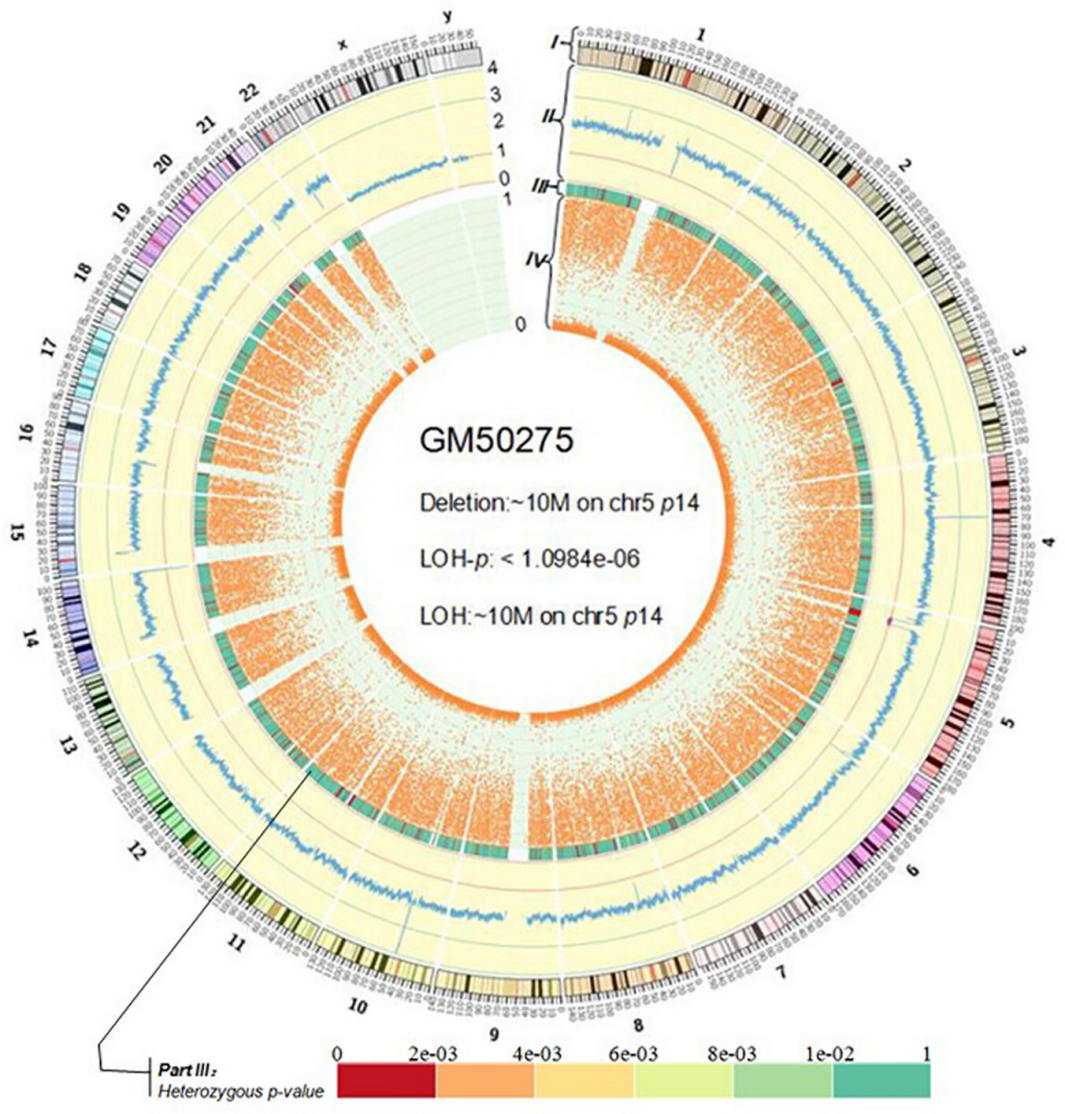
The Circos result of GM50275. In part II, CN can be predicted by dividing R_mi_ by 0.5 and a red line indicates a loss event and a green line displays a gain event.

In our study, Circos[26] is used to plot circular maps for a genome-wide view of relationships among genomic intervals. It depicts the details of whole-genome CNV and LOH features and is useful for a comprehension of the global picture. The figure is consisted of four parts from outside to inside: I) the chromosome ideograms in a pter-qter orientation, clockwise with the centromeres in red; II) the distribution of R_mi_ across whole genome with blue lines and the value of R_mi_ is from 4 to 0; III) the p-value views of heterozygous state; IV) the distribution of R_Het_ across whole genome with orange spots and the value of R_Het_ is from 1 to 0. As shown in Fig 6, one can see that there are 1 deletion and 1 LOH on chromosome 5p14 of the individual GM50275. Results for other individuals are shown in S4 Fig.

## Discussion

In this paper, we have proposed a novel integrated method, a selected target region approach (SeTR approach), for detecting genome-wide structural variations such as CNV, LOH and UPD. SeTRs are selected genome-wide with mean probe length of 150bp, the average distance among them of ~10kb, and the cumulative size of ~42Mb. Once sequenced to a certain depth, captured sequences of this set can be effectively used to detect structural variations and genomic aberrations for the entire genome. We also have developed a software package, ICLU, that uses statistical algorithms to detect of CNV, LOH and UPD for the SeTR sequencing approach. In addition, if one is only concerned about a specific CNV disease, or on a specific chromosome, or a certain collection of genomic hot spots, one can use a subset of our SeTRs within the interesting regions and our method will just work effectively as well.

With this selected target region approach, we don’t need to sequence the entire genome in order to detect CNVs. Our current approach only requires the sequencing of a fraction of the genome, about ~42Mb in size, or ~1.5% of the genome. In the extreme case, we can even lower the set to a minimal size of ~5Mb, or about ~0.17% of the genome, and still make correct predictions. With this approach, we can bring the coverage depth in the targeted regions much higher, and in the meantime, keep the overall cost of sequencing much smaller than that of a genome-wide sequencing approach. With the genomic sequencing cost dropping exponentially, our approach is a low-cost, high efficient method for detecting large structure aberrations such as CNV, LOH and UPD. It has the potential to displace other methods, such as the microarray based approaches, and the WGS methods.

At any specific location within genome, we perform noise reduction and signal smoothing using the medium coverage value for the entire collection of samples. This medium value matters a lot to us. Presumably, the healthy samples should far exceed diseased ones in a population for any specific region in question; otherwise one would be prone to make incorrect CNV calls. In the extreme, a sample size of 3 with at most 1 CNV in any specific genomic spot for the entire genome would be the absolute limit in applicability for our approach. In practice, for our method to make correct predictions, we would require a substantially larger collection of samples. Here we propose that a meaningful threshold of 8 samples as the minimum, and the samples should come from a random population.

Another limitation on our method concerns the detection of breaking points, or the exact CNV transition locations. We assume that each of our probes is located either entirely out of a CNV or entirely within. As we only sequence the genomic regions of SeTRs, a breaking point cannot be resolved beyond the two neighboring probes. What we do convey is to indicate that the two neighboring probes fall into two different CN regions. We also do not attempt to resolve any breaking point within a single probe, although in theory that can happen in ~1.5% cases (which is the coverage of our probes for the genome). So, our current limit of detection resolution is ~10K bases. A deeper read depth of SeTRs or a higher density of probes can improve the statistical power of CNV and LOH detection, and can also discover CNV events smaller in size. In contract, the approach of paired-end mapping[31,32] and *de novo* assembly of a genome[33] on WGS data would be more suitable to pinpoint breakpoints, to identify novel cross-chromosome events, and to completely characterizing the full spectrum of CNV and LOH.

In our study, 15 real samples captured by SeTRs kits and 8 simulated WGS samples are analyzed by ICLU. As the depth of coverage of target regions decreases gradually, the CNV results persist to be consistent with known karyotypes of real samples. True positive events of ~500kb CNVs in simulated samples have all been identified. Due to lack of parental information, LOHs and UPDs have not been validated. It is our understanding that LOH should happen with CN equals one. These events (CN = 1 and size>5Mb) in real samples are all correctly detected; and this reflects that our method for LOH detection is feasible. Moreover, when the R_Het_s of a genomic region presented is mainly around 0.5, such as dup(10) (q11.2q23.2) in GM05047 (S5 Fig), it indicates that the event’s CN may be changed to three. This appearance could also be used to support the accuracy in detecting CNV in ICLU.

In previous studies, people have developed CNV methods for CNVs in only exome regions[23,24,34]. We can combine these exome probe sets with our SeTR set. The combined probe set will be able to detect exon SNPs, indels, and identify genome-wide CNV and LOH for diseases. This approach may be financially meaningful, as we are only sequencing the minimum amount of the genome, yet we will have the ability to address the most urgent questions such as protein integrity and genomic integrity the same time.

## Conclusion

With the rapid development of sequencing technology and the fast decrease in price of NGS, detecting genomic alterations using a targeted sequencing strategy has the promises of high throughput and of low price. Price wise it should be less costly than both the microarray-based techniques and the WGS strategy. The targeted sequence data set offers a quick insight into CNV and LOH for specific diseases[35,36] or phenotypes in concern. Per conventions proposed in Itsara’s study, CN variants at the size larger than 500kb would usually be considered pathogenic in a clinical diagnostic setting[37]. This size fits well above our detection limit of 10kb. Therefore, our approach can detect all CNV events defined by current clinical standard. Our selected targeted region strategy, coupled with a much smaller size of sequenced genomic region and a decreased sequencing coverage depth, has tremendous financial advantages over other methods in clinics today. In addition, SeTRs sequencing can be combined with the sequencing of other genomic regions of interest, such as exomic regions to form an economic way of discovering genetic variations that have significant impact on human health[38].

## Materials and Methods

### Designing SeTRs

Genomic regions with extreme GC content (high or low) or with high polymorphism rates negatively impact their PCR or target capture efficiency[23,39]. In some previous studies, GC-content adjustment and mappability corrections have been applied in computation to remove experimental bias[22,40–42]. In our study, we select special target regions, called evenly distributed selected target regions (SeTRs) to avoid coverage bias due to sequence content. We select candidate SeTRs using the following criteria: (i) the uniqueness and stability properties of the region. We require less polymorphism and a modest GC content; (ii) a small number of sparse SNPs within to detect LOH, and that these SNPs are present with high frequency in population; (iii) the probes are relatively uniform in distribution within the entire genome. Each target region is captured by one and only one probe.

The set of SeTR locations across the entire genome has been selected by the following steps:

- SNPs set1: Based on SNPs database of the 1000 Genome Project (web: ftp://ftp.ncbi.nih.gov/1000genomes/ftp/release/), SNPs with allele frequency (AF) ranging 10% to 90% in population have been retained as candidates. A portion of clustered SNPs, i.e. those located within the neighborhood of 100bp of another selected SNP, are removed.
- SNPs set2: SNPs set1 is filtered further using the reference genome. We construct short sequences around each SNP of 100 bases in length, using 50bp upstream and 50bp downstream from the SNP site. These short sequences are then mapped to the reference genome by BLAST[43]. If the alignment for a short sequence shows no mismatch for the best mapping and within less than 5% mismatch by the second best mapping, the corresponding SNP is retained in SNPs set2.
- SNPs set3: Based on SNPs set2, the SNPs which are evenly distributed on the whole genome are selected as final selected SNPs. In our study, the ideal physical distance between two adjacent SNPs is set at 10k base. If an interval of 10k size contains more than one SNP in SNPs set2, only one is kept. SNPs set3 may contain large gaps within the neighboring SNPs.
- Final set for probe locations: For SNPs set3, if the physical distance of two adjacent SNPs was more over 10k base, one or more selected target locations, selected to be evenly distributed within this gap region, are inserted. These additional locations make our collection of SeTR locations complete. We now have achieved a set of locations that are relatively evenly distributed across of the entire genome.

The typical gap size between two neighboring probe locations is around 10k base. The location may be a SNP location from the 1000 Genome Project, or it may simply be a sequenced location within the reference genome. In location selection, given the requirements of achieving a relative evenness in distribution, but not an absolute evenness, we do have the freedom of avoiding simple repetitive regions, and the regions with extreme GC values.

### The source of samples and simulated data

The cell lines of 13 samples have been bought from The Coriell Institute, containing 2 aneuploid samples and 11 micro deletion or duplication samples. All of their karyotype results and catalogue ID (S6 Table) can be found from the webpage (http://ccr.coriell.org/Default.aspx?public=true) using GM id. In addition, the YH sample, a healthy Chinese individual, and the HG00537 sample (www.1000genomes.org) with normal karyotype and 5 DNA samples from aborted fetuses were used in our evaluation of the method. We also used simulated data for evaluation. A collection of 8 WGS data were generated via computer simulation, with the samples containing a total of 9 true CNV events.

### Sequencing read mapping

After the whole genome shotgun library was constructed, the target PCR products captured by SeTRs kits were sequenced on the Illumina HiSeq2000 sequencer following manufacturer’s instructions. Raw sequencing data was filtered by some bioinformatics screens (screening out low quality reads and contaminated reads by using adapter and bacteria sequences). The remaining data were mapped to the reference human genome (hg19, Build 37.1) using BWA[44] with default parameters. We then process the alignments by using SAMtools[45] to remove PCR duplications. We also run local realignment around indels and base quality score recalibration employing the Genome Analysis Tool Kit (GATK) software[46].

### Genome-wide CNV screening

According to re-alignment results, the first step was to calculate the depth of coverage in every target region, denoted as TD_i_ (i.e. *Target Depth for region i*). Then, each TD_i_ was corrected to TD_mi_ using moving average in order to ensure the continuity and stability of fluctuation in adjacent regions. TD_mi_ was then normalized by dividing by $\bar{{TD}_{mi}}$ (the average TD_mi_ of all target regions for all autosomal chromosomes) to get the depth coefficient R_i_ and then divide R_i_ by the median R_i_ from multiple samples’ target region *i* to get R_mi_.

The relevant computation formula is as follow:

$$TD_{i}=T_{i}base/T_{i}len$$ 1

$$TD_{mi}=(\sum_{i+n}^{i}TD_{i})/(n+1),n\geq 10$$ 2

$$\bar{TD}=(\sum_{i+n}^{i}TD_{i})/(n+1)$$ 3

$$\bar{TD_{m}}=(\sum_{i+n}^{i}TD_{mi})/(n+1)$$ 4

$$R_{i}=TD_{mi}/\bar{TD_{m}}$$ 5

$$preR_{i}=TD_{i}/\left(\left(\sum_{N}^{1}TD_{i}\right)/N\right)$$ 6

Note: *T*_*i*_ *base* was the number of aligned bases in the region *i* and *T*_*i*_ *len* was its length.

In theory, all R_mi_ from multiple samples in the specific region *i* follow normal distribution. For a given test sample in region *i*, T-test was adapted to detect a CNV signal using parameters estimated from the collection of samples.

$$t=\frac{\left(\bar{R_{mi1}}-\bar{R_{mi2}})-(\mu_{1}-\mu_{2}\right)}{\sqrt{\frac{(n_{1}-1)S_{1}^{2}+(n_{2}-1)S_{2}^{2}}{n_{1}+n_{2}-2}(\frac{1}{n_{1}}+\frac{1}{n_{2}})}}∼t_{n_{1}+n_{2}-2}$$ 7

When the number of test samples was 1 and the number of multiple samples was n, under the condition of the same R_mi_ distribution in each population, formula (7) can be simplified to:

$$t=\frac{R_{mi\_test}-\bar{R_{mi\_multiple}}}{\sqrt{S_{multiple}^{2}(1+\frac{1}{n})}}$$ 8

According to formula (8), a T-score and a p-value of each region *i* can be calculated. A region with p-value less than 0.05 was considered as a CNV signal in our study; and copy number for the region was simply predicted by dividing R_m_ by 0.5 and taken it to the nearest integer (the nearest integer function):CN = int(R_mi_/0.5). Based on the p-value from T-test of a target region, a pseudo signal was appended to each probe to indicate whether it was implicated in the CNV region for the next step. Then, neighboring target regions having same copy numbers will be merged together to form larger intervals across the entire chromosome. Here is an idea on merging neighboring target regions into large intervals: A continuous 4 target regions was set as the minimum interval size if they had the same direction of copy number change (T-score <0 or >0) and 3 of their p-values were less than the first threshold value (i.e. 0.05, common threshold set for tests of significance), and the fourth p-value should not exceed a second threshold (set at 0.2,i.e. Four times the first threshold value). Once meeting these condition, all continuous 4 target regions would be mark ‘-’ or ‘+’ as a pseudo signal. With the same pseudo signal, the two sets of {*i*..*i* + *k*; *k* ≥ 3, *i* ≥ 1; *i*, *k* ∈ *n*} and {*j*..*j + l*; *l* ≥ 3, *j* ≥ *i* + *k*; *j*, *l* ∈ *n*} that were separated by less than 5 target regions, i.e. *j*−(*i* + 3) ≤ 5, would be merged as a single contiguous region of {*i*..*j* + *l*}. By analogy, for the merge large sets of {*i*..*N*; *N* ≥ 4, *i* ≥ 1; *N*, *i* ∈ *n*}, T-test was applied again between the test sample and the multiple samples using R_mi_ for the regions of {*i*..*N*; *N* ≥ 4, *i* ≥ 1; *N*, *i* ∈ *n*} as formula (9) and (10) showed. After this heuristic approach, the boundary, size and CN of {*i*..*N*; *N* ≥ 4, *i* ≥ 1; *N*, *i* ∈ *n*} would be reported.

$$Z_{i}=R_{mi\_test}-\bar{R_{mi\_multiple}}$$ 9

t=∑NiZi(N−i+1)SZiN−i∼t(N−i) 10

### Genome-wide LOH and UPD screening

SNP positions with allele frequencies between 0.1 and 0.9 in the 1000 Genome SNPs Database in the target regions of samples are used to detect heterozygosity. For the position *i*, the B-allele count is the number of reads with non-reference calls at this position. The B-Allele Frequency, aka BAF, is the B-allele count divided by the total number of reads mapped to position *i*. R_Het_, the heterozygosity advantage rate of the position *i*, is calculated by formula (11) and it represents the heterozygous state of position *i*.

$$R_{Het}=\text{min}\left\{\frac{BAF}{1-BAF},\frac{1-BAF}{BAF}\right\},R_{Het}\subseteq [0,1]$$ 11

If position *i* appears to be an absolute heterozygous state, its R_Het_ would be 1. On the contrary, when the R_Het_ equals 0, position *i* is completely homozygous. An F-test has been applied to detect LOH in whole genome using SD of R_Het_s as follow: In the test sample, a subset of R_Het_s, has been constructed from the position i to j, denoted by *T*_*ij*_ = {*R*_*Het_i*_, *R*_*Het_i*+1_,…, *R*_*Het_j*_; *i*, *j* ∈ *n*}. The corresponding, $M_{ij}=\left\{{\tilde{R}}_{Het\_i},{\tilde{R}}_{Het\_i+1},\ldots ,{\tilde{R}}_{Het\_j};i,j\in n\right\}$ could be identified from multiple samples, here ${\tilde{R}}_{Het\_i}$ denotes the median value of R_Het_i_s for all samples at the position i. Standard deviation (SD) of T_ij_ was compared with SD of M_ij_ by F-test to accept the null hypothesis (H_0_) or the alternative hypothesis (H_A_) under the threshold of the p-value 0.01. If the p-value of T_ij_ is lower than 0.01, H_A_ is accepted. It means that the subset of T_ij_ has lost heterozygosis comparing with the multiple samples. See formulas below for calculation details.

$$S_{test}^{2}=\frac{{\sum_{i\leq r\leq j}\left(R_{test\_r}-\bar{R_{test\_r}}\right)}^{2}}{n-1}$$ 12

$$S_{mul}^{2}=\frac{\sum_{i\leq r\leq j}{\left(R_{mul\_r}-\bar{R_{mul\_r}}\right)}^{2}}{n-1}$$ 13

$$S_{\text{max}}^{2}=\text{max}\left\{S_{test}^{2},S_{mul}^{2}\right\},S_{\text{min}}^{2}=\text{min}\left\{S_{test}^{2},S_{mul}^{2}\right\}$$ 14

$$F_{upper}=\frac{S_{\text{max}}^{2}}{S_{\text{min}}^{2}},df_{test}=df_{mul}=n-1\;,F_{under}=\frac{S_{\text{min}}^{2}}{S_{\text{max}}^{2}},df_{test}=df_{mul}=n-1$$ 15

$$p-value=p_{upper}+(1-p_{under})$$ 16

We scan the continuous sets of {*T*_*k*_, *T*_*k* + 1_,…,*T*_*l*_; *k*, *l* ∈ *n*; *l*−*k* ≥ 3}, and initiate a LOH interval if p-value is less than 0.01 for 3 continuous probes. Thus, our minimal LOH event has interval size spanning 3 probes. We extend this LOH by adding neighboring probes with small p-values. We allow the continuous expansion of LOH region if only one probe has p-value greater than 0.01 but the mean p-value for the entire region {*T*_*k*_, *T*_*k* + 1_,…,*T*_*l*_; *k*, *l* ∈ *n*; *l*−*k* ≥ 3} is still less than 0.1. In another word, if the p-value of {*T*_*k*_, *T*_*k* + 1_,…,*T*_*l*_; *k*, *l* ∈ *n*; *l*−*k* ≥ 3} of the extended region is smaller than 0.01, *HA*: *σ*_*test*_ ≠ *σ*_*mul*_ is accepted and that {*T*_*k*_, *T*_*k* + 1_,…,*T*_*l*_; *k*, *l* ∈ *n*; *l*−*k* ≥ 3} is predicted as a larger LOH.

The isodisomy of UPD occurs when a person receives two copies of a part or entire chromosome from one parent because of a duplication event. Integrating the results from genome-wide CNV computation and heterozygosis screening, the isodisomy can be evaluated by applying this definition. If a segment presents that an LOH event has happened and the copy number is normal at the same time, we can conclude that the segment is an isodisomy.

## Supporting Information

S1 Fig The characteristics of SeTRs on whole genome. (A) The distribution of the SeTRs probe length; (B) The distribution of the gap sizes of adjacent probes in SeTRs.
(TIF)

S2 Fig The CNV results for eight simulated WGS samples using ICLU pipeline. From outside to inside, the turn is from sample 1 to sample 8 and the detected CNV events are presented with purple solid line.
(TIF)

S3 Fig The performance of ICLU on ~42Mb SeTRs with the decrease of depth-of-coverage. (TIF)

S4 Fig The Circos results of fifteen real samples. (TIF)

S5 Fig The distribution of R_Het_s (green spots) across chromosome 10 on GM05047. When the CN of a fragment with heterozygosity is three, the sets of R_Het_s of the fragment cluster is around 0.5 (between two red dotted lines). Following this observation, R_Het_ can also be used to predict CNV events, or be used to verify the accuracy of a CNV prediction.
(TIF)

S1 Table The SeTRs statistics by chromosome. (DOCX)

S2 Table The performance of ICLU and CONTRA on a 30X coverage of simulated WGS data set. (DOCX)

S3 Table The performance of ICLU for detecting CNV with~42Mb and~5Mb size of SeTRs. (DOCX)

S4 Table The performance of ICLU for detecting CNV with different depth on 15 real samples’ SeTRs data. (XLS)

S5 Table The CNV analysis of SeTRs with ICLU algorithm and WGS method on the five abortion samples. (DOCX)

S6 Table Catalogue number of the 13 cell line samples bought from Coriell Institute. (DOCX)

S1 Text No competing Interest declared by Y. Tom Tang. (PDF)
