# Correction: Identifying Human Genome-Wide CNV, LOH and UPD by Targeted Sequencing of Selected Regions

**DOI:** 10.1371/journal.pone.0132537

**Published:** 2015-07-02

**Authors:** The PLOS ONE Staff

## Notice of Republication

This article was republished on June 12, 2015 to correct the omission of Corresponding Author Yu Wang and his affiliation from the Author byline. The publisher apologizes for the error. Please download this article again to view the correct version. The originally published, uncorrected article and the republished, corrected article are provided here for reference.

## Supporting Information

S1 File Originally published, uncorrected article. (PDF)

S2 File Republished corrected article. (PDF)

## References

[1] Wang Y, Li W, Xia Y, Wang C, Tang YT, Guo W, et al (2015) Identifying Human Genome-Wide CNV, LOH and UPD by Targeted Sequencing of Selected Regions. PLoS ONE 10(4): e0123081 doi: 10.1371/journal.pone.0123081 25919136 10.1371/journal.pone.0123081 PMC4412667

