# Supplementary figures and images for: Identifying Human Genome-Wide CNV, LOH and UPD by Targeted Sequencing of Selected Regions
Source: PLoS One. 2015 Apr 28;10(4):e0123081. doi: 10.1371/journal.pone.0123081 (PMC4412667; doi:10.1371/journal.pone.0123081)

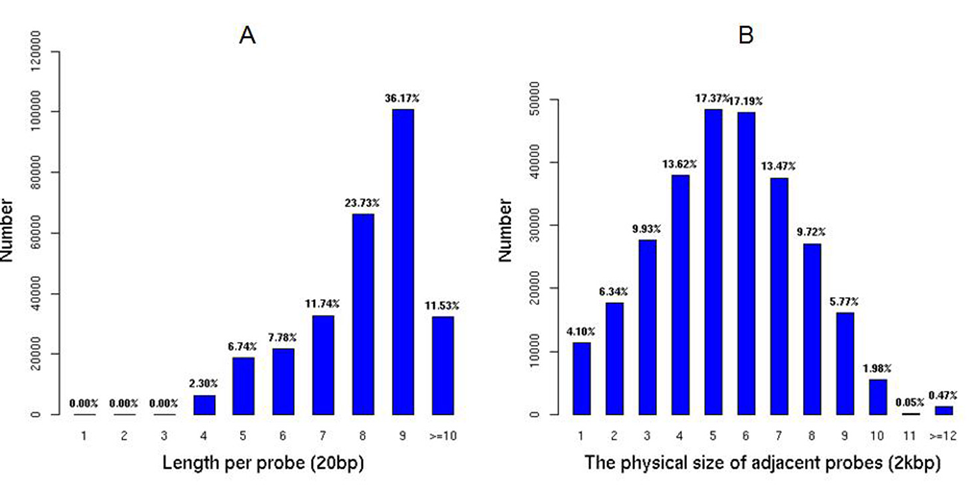

Supplement: S1 Fig — (A) The distribution of the SeTRs probe length; (B) The distribution of the gap sizes of adjacent probes in SeTRs. (TIF) [file pone.0123081.s001.tif]

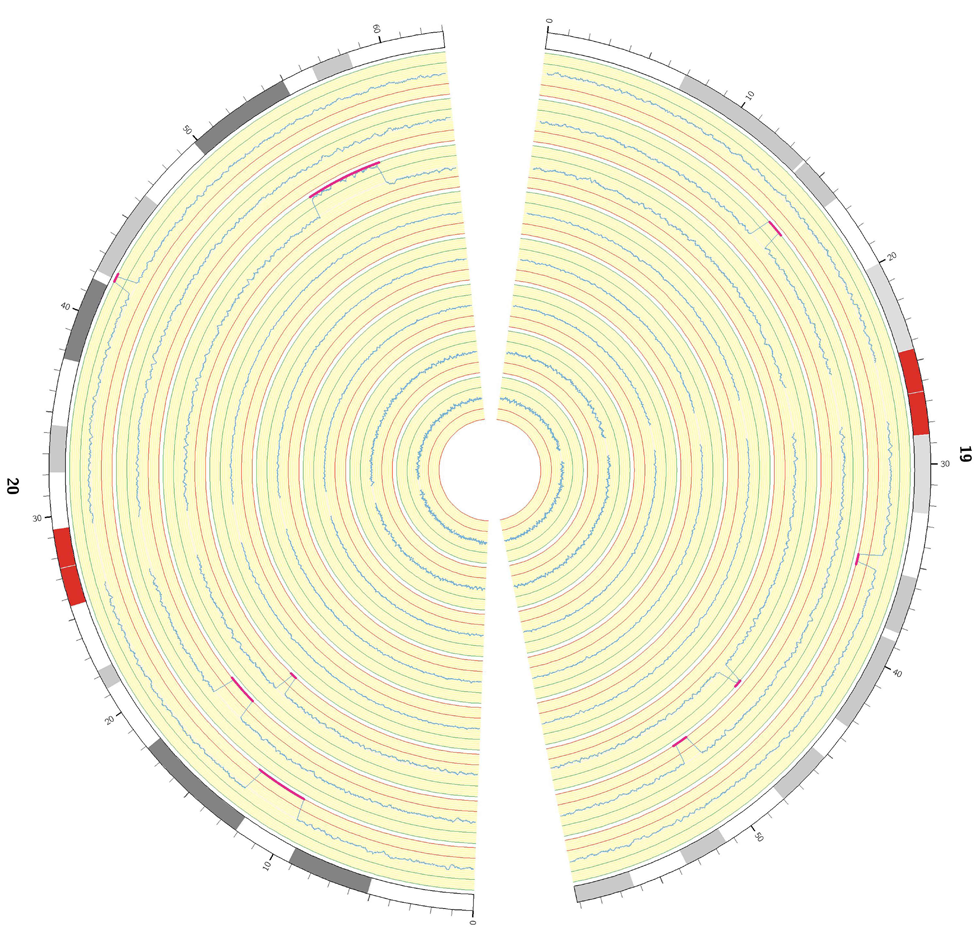

Supplement: S2 Fig — From outside to inside, the turn is from sample 1 to sample 8 and the detected CNV events are presented with purple solid line. (TIF) [file pone.0123081.s002.tif]

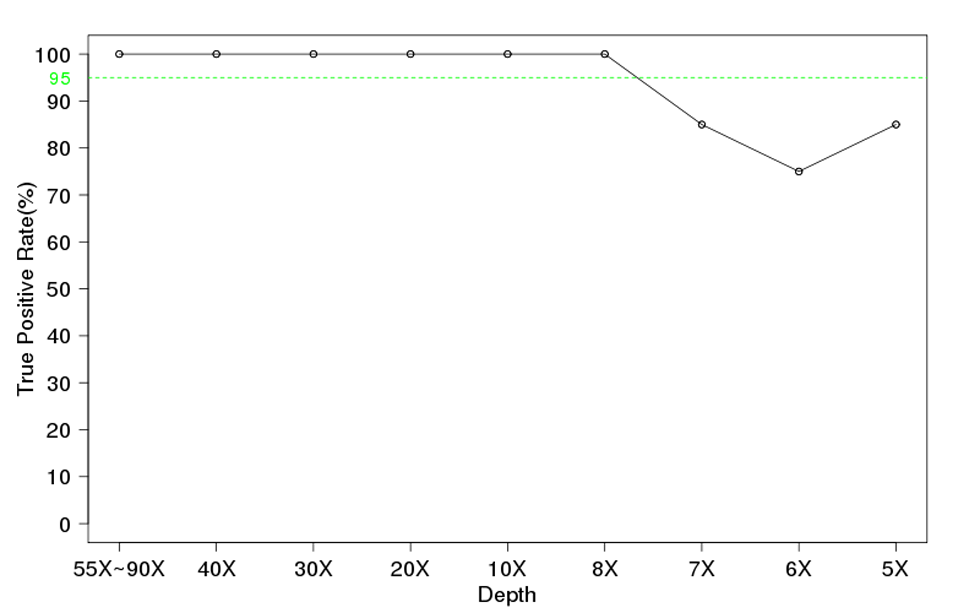

Supplement: S3 Fig — (TIF) [file pone.0123081.s003.tif]

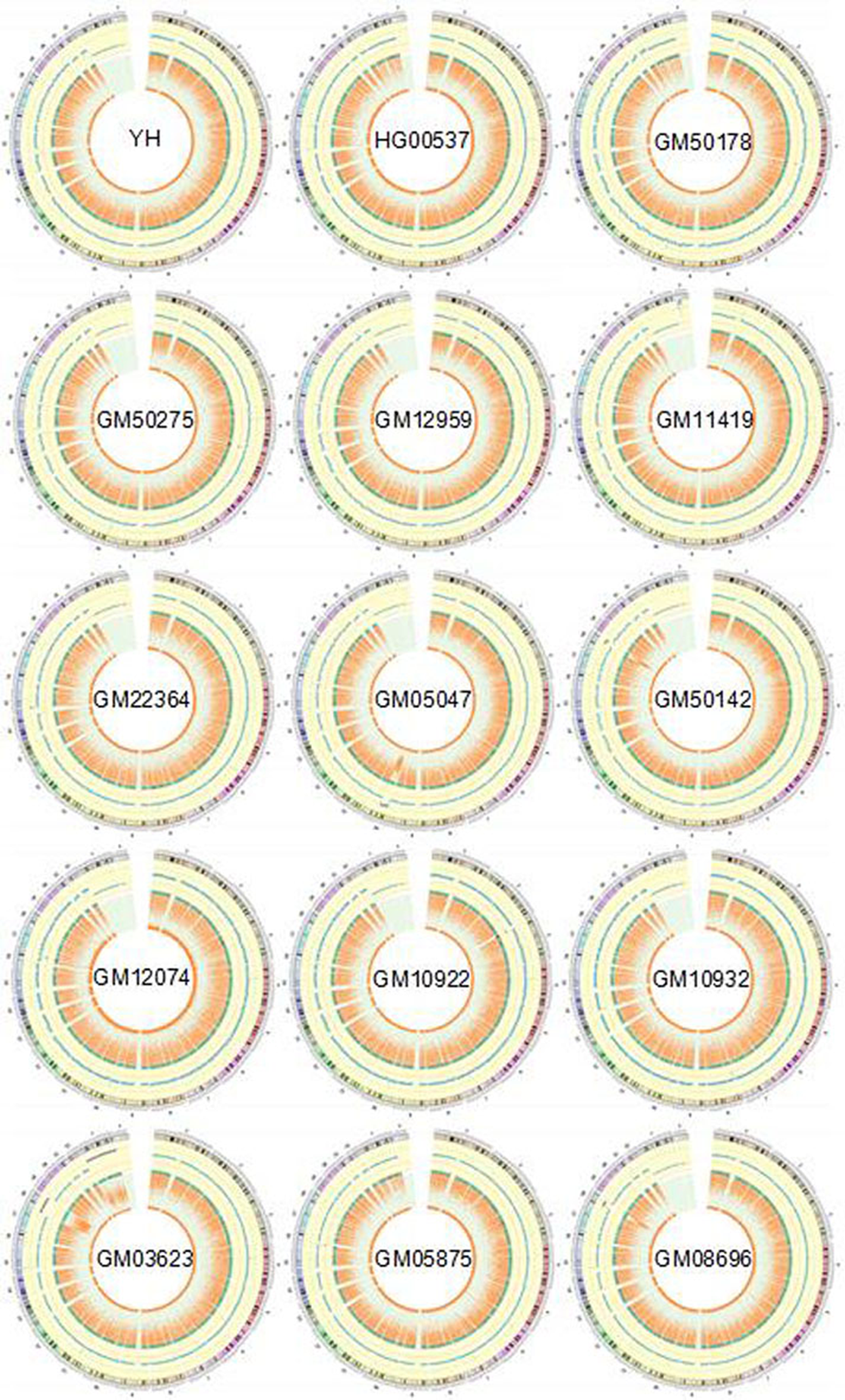

Supplement: S4 Fig — (TIF) [file pone.0123081.s004.tif]

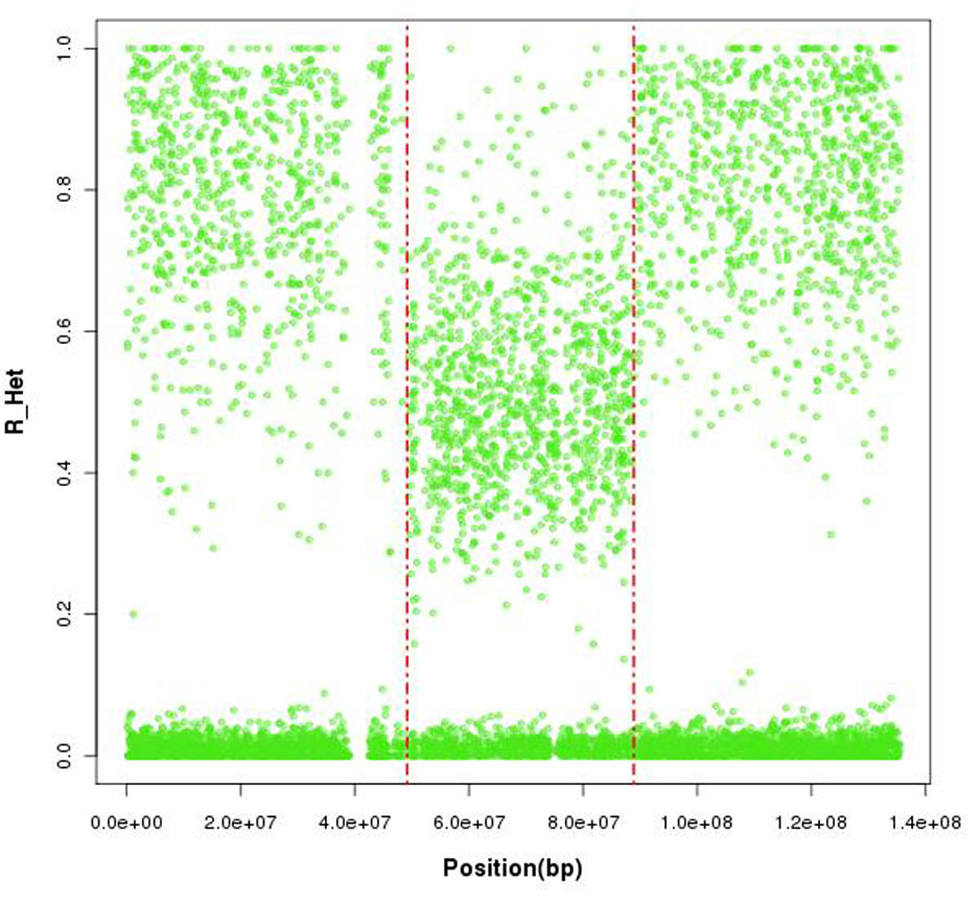

Supplement: S5 Fig — When the CN of a fragment with heterozygosity is three, the sets of RHets of the fragment cluster is around 0.5 (between two red dotted lines). Following this observation, RHet can also be used to predict CNV events, or be used to verify the accuracy of a CNV prediction. (TIF) [file pone.0123081.s005.tif]
