# Supplementary material for: Identifying Human Genome-Wide CNV, LOH and UPD by Targeted Sequencing of Selected Regions
Source: PLoS One. 2015 Apr 28;10(4):e0123081. doi: 10.1371/journal.pone.0123081 (PMC4412667; doi:10.1371/journal.pone.0123081)
Supplement: S1 Table — (DOCX) [file pone.0123081.s006.docx]

**Table S1.** The SeTRs statistics by chromosome.

| **Chromosome** | **Number** | **Average length of probes** | **Average gap size of adjacent probes** |
| --- | --- | --- | --- |
| **chr1** | 21938 | 150 | 6821 |
| **chr2** | 23407 | 151 | 5715 |
| **chr3** | 19563 | 150 | 5920 |
| **chr4** | 18699 | 149 | 6096 |
| **chr5** | 17551 | 150 | 6340 |
| **chr6** | 16305 | 150 | 6727 |
| **chr7** | 14983 | 149 | 7132 |
| **chr8** | 14222 | 150 | 6844 |
| **chr9** | 10865 | 150 | 10168 |
| **chr10** | 12825 | 150 | 7390 |
| **chr11** | 12948 | 149 | 7212 |
| **chr12** | 13110 | 149 | 6999 |
| **chr13** | 9583 | 149 | 7437 |
| **chr14** | 8804 | 149 | 7606 |
| **chr15** | 7716 | 150 | 8662 |
| **chr16** | 7364 | 150 | 10141 |
| **chr17** | 7485 | 148 | 8828 |
| **chr18** | 7431 | 151 | 8370 |
| **chr19** | 5521 | 144 | 8972 |
| **chr20** | 5957 | 152 | 8872 |
| **chr21** | 3353 | 149 | 10433 |
| **chr22** | 3309 | 150 | 9355 |
| **chrX** | 14547 | 145 | 7086 |
| **chrY** | 1314 | 130 | 42448 |
