# Supplementary material for: Identifying Human Genome-Wide CNV, LOH and UPD by Targeted Sequencing of Selected Regions
Source: PLoS One. 2015 Apr 28;10(4):e0123081. doi: 10.1371/journal.pone.0123081 (PMC4412667; doi:10.1371/journal.pone.0123081)
Supplement: S2 Table — (DOCX) [file pone.0123081.s007.docx]

**Table S2.** The performance of ICLU and CONTRA on a 30X coverage of simulated WGS data set.

| **Information** | **Sample** | **Chr** | **Start** | **End** | **Size(M)** | **CN** | **Sensitivity** | **Specificity** |
| --- | --- | --- | --- | --- | --- | --- | --- | --- |
| **Simulation** | Sample1~5 | Normal | | | | | NA | NA |
|  | Sample6 | chr20 | 15000000 | 15500000 | 0.5- | 0 |  |  |
|  |  | chr20 | 53000000 | 58000000 | 5+ | 4 |  |  |
|  |  | chr19 | 45000000 | 45500000 | 0.5+ | 4 |  |  |
|  | Sample7 | chr20 | 16000000 | 18000000 | 2- | 0 |  |  |
|  |  | chr19 | 15000000 | 16000000 | 1+ | 4 |  |  |
|  |  | chr19 | 50000000 | 51000000 | 1- | 0 |  |  |
|  | Sample8 | chr20 | 10000000 | 13000000 | 3- | 0 |  |  |
|  |  | chr20 | 42000000 | 42450000 | 0.45+ | 4 |  |  |
|  |  | chr19 | 35000000 | 35600000 | 0.6- | 0 |  |  |
| **Total CNV** | 9 true positive CNVs | | | | | |  |  |
| **ICLU** | Sample1~5 | Normal | | | | | 100% | 100% |
|  | Sample6 | chr20 | 15007645 | 15492763 | 0.49- | 0 |  |  |
|  |  | chr20 | 53000101 | 57995856 | 5+ | 4 |  |  |
|  |  | chr19 | 45003283 | 45496699 | 0.49+ | 4 |  |  |
|  | Sample7 | chr20 | 15983112 | 17992034 | 2- | 0 |  |  |
|  |  | chr19 | 15009149 | 16000694 | 0.99+ | 4 |  |  |
|  |  | chr19 | 50000342 | 50998777 | 1- | 0 |  |  |
|  | Sample8 | chr20 | 10018089 | 12995181 | 2.98- | 0 |  |  |
|  |  | chr20 | 42008751 | 42442770 | 0.43+ | 4 |  |  |
|  |  | ch19 | 35004532 | 35595304 | 0.59- | 0 |  |  |
| **Total CNV** | 9 true positive CNVs, 0 false positive CNVs | | | | | |  |  |
| **CONTRA** | Sample1~5 | Normal | | | | | 88.9% | 66.7% |
|  | Sample6 | chr20 | 15007645 | 15492763 | 0.49- | NA |  |  |
|  |  | chr19 | 45003283 | 45496699 | 0.49+ | NA |  |  |
|  | Sample7 | chr20 | 16009467 | 17992034 | 1.98- | NA |  |  |
|  |  | chr19 | 15009149 | 15993267 | 0.98+ | NA |  |  |
|  |  | chr19 | 50000342 | 50998777 | 1- | NA |  |  |
|  | Sample8 | **chr20** | **63704** | **9990568** | **9.93-** | NA |  |  |
|  |  | chr20 | 10007121 | 12995181 | 2.99- | NA |  |  |
|  |  | **chr20** | **19869958** | **35830028** | **15.96+** | NA |  |  |
|  |  | chr20 | 42008751 | 42442770 | 0.43+ | NA |  |  |
|  |  | **chr19** | **3430053** | **31917721** | **28.49+** | NA |  |  |
|  |  | chr19 | 35004532 | 35595304 | 0.59- | NA |  |  |
| **Total CNV** | 8 true positive CNVs, 3 false positive CNVs | | | | | |  |  |

Note:"-" indicates "Deletion","+" indicates "Duplication"
