# Supplementary material for: Identifying Human Genome-Wide CNV, LOH and UPD by Targeted Sequencing of Selected Regions
Source: PLoS One. 2015 Apr 28;10(4):e0123081. doi: 10.1371/journal.pone.0123081 (PMC4412667; doi:10.1371/journal.pone.0123081)
Supplement: S3 Table — (DOCX) [file pone.0123081.s008.docx]

**Table S3.** The performance of ICLU for detecting CNV with ~42Mb and ~5Mb size of SeTRs.

| **Coverage Depth** | **SeTRs(Mb)** | **Sensitivity** | **Specificity** | **Accuracy** | **PPV** | **NPV** |
| --- | --- | --- | --- | --- | --- | --- |
| **40X** | 42 | 100% | 100% | 100% | 100% | 100% |
| **30X** | 42 | 100% | 100% | 100% | 100% | 100% |
| **20X** | 42 | 100% | 100% | 100% | 100% | 100% |
| **10X** | 42 | 100% | 100% | 100% | 100% | 100% |
| **40X** | 5 | 100% | 100% | 100% | 100% | 100% |
| **30X** | 5 | 100% | 100% | 100% | 100% | 100% |
| **20X** | 5 | 100% | 100% | 100% | 100% | 100% |
| **10X** | 5 | 100% | 100% | 100% | 100% | 100% |

Sensitivity= TP / (TP + FN)

Specificity= TN / (FP + TN)

Accuracy (ACC) =TP+TN/Total population

Positive predictive value (PPV) = TP / (TP + FP)

Negative predictive value (NPV) = TN / (FN + TN)
