# Supplementary material for: Identifying Human Genome-Wide CNV, LOH and UPD by Targeted Sequencing of Selected Regions
Source: PLoS One. 2015 Apr 28;10(4):e0123081. doi: 10.1371/journal.pone.0123081 (PMC4412667; doi:10.1371/journal.pone.0123081)
Supplement: S5 Table — (DOCX) [file pone.0123081.s010.docx]

**Table S5.** The CNV analysis of SeTRs with ICLU algorithm and WGS method on the five abortion samples.

| **Sample** | **SeTRs with ICLU** | **WGS method** |
| --- | --- | --- |
| **PDT13IMS00066** | 46,XX,del(1)(p36.22-36.33),dup(1)(q32.2-44) | 46,XX,del(1)(p36.22-36.33),dup(1)(q32.2-44) |
| **PDT13IMS00094** | 46,XY,dup(3)(q21.3-29),del(11)(q23.3-25),dup(13)( q12.12) | 46,XY,dup(3)(q21.3-29),del(11)(q23.3-25),dup(13)(q12.12) |
| **PDT13IMS00160** | 45,XO,del(6)(q26-27),dup(10)(q21.1-26.3) | 45,XO,del(6)(q26-27),dup(10)(q21.1-26.3) |
| **IMS13S00027** | 46,XX,del(6)(q15-16.1) | 46,XX,del(6)(q15-16.1) |
| **IMS13S00028** | 46,XX,dup(18)(q23) | 46,XX,dup(18)(q23) |
