# Supplementary material for: Identifying Human Genome-Wide CNV, LOH and UPD by Targeted Sequencing of Selected Regions
Source: PLoS One. 2015 Apr 28;10(4):e0123081. doi: 10.1371/journal.pone.0123081 (PMC4412667; doi:10.1371/journal.pone.0123081)
Supplement: S6 Table — (DOCX) [file pone.0123081.s011.docx]

**Table S6.** Catalogue number of the 13 cell line samples bought from Coriell Institute

| **Sample** | **Catalog ID** |
| --- | --- |
| **GM50178** | GM50178 |
| **GM50275** | GM50275 |
| **GM12959** | GM12959 |
| **GM11419** | GM11419 |
| **GM22364** | GM22364 |
| **GM05047** | GM05047 |
| **GM50142** | GM50142 |
| **GM12074** | GM12074 |
| **GM10922** | GM10922 |
| **GM10932** | GM10932 |
| **GM03623** | GM03623 |
| **GM05875** | GM05875 |
| **GM08696** | GM08696 |
