# Supplementary material for: Identifying Human Genome-Wide CNV, LOH and UPD by Targeted Sequencing of Selected Regions
Source: PLoS One. 2015 Apr 28;10(4):e0123081. doi: 10.1371/journal.pone.0123081 (PMC4412667; doi:10.1371/journal.pone.0123081)
Supplement: S1 Text — (PDF) [file pone.0123081.s012.pdf]

PLOS Editorial Board

11/3/2014

To Whom It May Concern:

Concerning the submitted manuscript entitled: "Identifying Human Genome-wide CNV, LOH and UPD by Targeted Sequencing of Selected Regions", I, the undersigned, declare that I have no competing interests for this manuscript and the reported research work here.

Sincerely yours,

Yuanhua Tom Tang, PhD

Director of Bioinformatics
Complete Genomics, Inc.
